# Role of the Hippo Pathway in Fibrosis and Cancer

**DOI:** 10.3390/cells8050468

**Published:** 2019-05-16

**Authors:** Cho-Long Kim, Sue-Hee Choi, Jung-Soon Mo

**Affiliations:** 1 Department of Biomedical Sciences, Cancer Biology Graduate Program, Ajou University Graduate School of Medicine, Suwon 16499, Korea; cholong92@ajou.ac.kr (C.-L.K.); tngml1984@ajou.ac.kr (S.-H.C.); 2 Genomic Instability Research Center (GIRC), Ajou University School of Medicine, Suwon 16499, Korea

**Keywords:** YAP, TAZ, MST1/2, LATS1/2, myofibroblast, ECM, fibrosis, EMT, cancer

## Abstract

The Hippo pathway is the key player in various signaling processes, including organ development and maintenance of tissue homeostasis. This pathway comprises a core kinases module and transcriptional activation module, representing a highly conserved mechanism from *Drosophila* to vertebrates. The central MST1/2-LATS1/2 kinase cascade in this pathway negatively regulates YAP/TAZ transcription co-activators in a phosphorylation-dependent manner. Nuclear YAP/TAZ bind to transcription factors to stimulate gene expression, contributing to the regenerative potential and regulation of cell growth and death. Recent studies have also highlighted the potential role of Hippo pathway dysfunctions in the pathology of several diseases. Here, we review the functional characteristics of the Hippo pathway in organ fibrosis and tumorigenesis, and discuss its potential as new therapeutic targets.

## 1. Introduction

The Hippo pathway is an evolutionarily conserved signaling transduction nexus that influences a wide range of biological processes during the growth and development of tissues and organs. This signaling pathway has recently been recognized as a master regulator of the malignant progression of many cancers by regulating cell proliferation and stem/progenitor-cell expansion [1]. Genetic studies in *Drosophila* provided the first identification of the Warts (Wts) as tumor suppressors [2,3].

Subsequent molecular studies further defined the details of core components of the Hippo pathway and their cellular functions, including cell growth, proliferation, survival, and organ-size control, demonstrating high levels of conservation from *Drosophila* to mammals. The Hippo pathway consists of a core kinases module and a transcriptional activation module. In *Drosophila*, the Hippo pathway includes a kinase cascade of Ste20-like kinase Hippo (Hpo) and NDR family kinase Wts [2,3,4,5,6,7,8]. Hpo forms a complex with the scaffolding protein Sav to phosphorylate and activate Wts, which physically associates with its regulatory protein Mob as Tumor Suppressor (Mats) [9,10,11]. Phosphorylated and activated Wts then inactivates Yorkie (Yki) via phosphorylation at serine residues [12], allowing it to interact with 14-3-3 and thereby accumulate in the cytosol [12,13,14,15]. The core components of the mammalian Hippo pathway include a kinase cascade of mammalian sterile 20-like kinase 1/2 (MST1/2) and large tumor suppressor kinase 1/2 (LATS1/2) [8,16,17,18]. MST1/2 and its regulatory protein SAV1 physically interact with and phosphorylate LATS1/2 kinase. Active LATS1/2 kinase in complex with Mob1 phosphorylates and inactivates the transcriptional co-activator Yes-associated protein (YAP) and transcriptional co-activator with a PDZ-binding motif (TAZ) to influence their subcellular localization in a 14-3-3-dependent manner [12,13,14,15,19]. YAP has also been identified as YES1-interacting protein, a tyrosine kinase, which is a key downstream effector of the Hippo pathway to function as a transcriptional co-activator [20]. TAZ (encoded by *WWTR*) is composed of a WW domain and PDZ-binding motif was demonstrated to play a key role as another mediator of the Hippo pathway similar to its paralog YAP [21]. Based on studies in *Drosophila* and mammals, the Hippo pathway kinase LATS1/2 regulates YAP/TAZ activity and their subcellular localization by direct phosphorylation of five consensus HX(R/H/K)XX(S/T) motifs [22,23,24,25]. Phosphorylated YAP/TAZ associate with 14-3-3 protein, leading to their cytoplasmic accumulation and the consequent loss of transcriptional co-activator function. Cytosolic YAP/TAZ are further phosphorylated by CK1 and LATS1/2, and are then rapidly destroyed by the ubiquitin-proteasome and lysosome system [26]. Conversely, unphosphorylated YAP/TAZ translocate to the nucleus and bind to the TEAD family transcription factors to activate the transcription of target genes that promote cell proliferation and inhibit apoptosis [27,28]. Besides TEADs, YAP/TAZ can interact with several other proteins, including Smad, Runx family, p73, ErbB4, Pax3, TTF1, PPARγ, and TBX5, which are responsible for transcriptional activation, and VGLL4, which is responsible for transcriptional repression [29].

Despite these functional similarities of YAP and TAZ, they nevertheless differ in their physiological and developmental functions. Much progress has been made in identifying the multiple new regulators of the Hippo pathway. Moreover, accumulating evidence, both physiological and pathological, points to a role of the central upstream Hippo kinase cascade in regulating the downstream effectors YAP/TAZ that sense extracellular environment signals, and integrate intracellular signaling to maintain a variety of biological functions, including tissue homeostasis and cancer [30,31,32]. Here, we review this research progress of the specific roles of YAP/TAZ to provide new insight into these important mediators of the Hippo pathway as effectors of fibrosis and cancer progression.

## 2. The Hippo Pathway in Organ Fibrosis

Myofibroblasts are responsible for fibrogenesis and produce a series of extracellular matrix (ECM) components such as collagens, laminins, and fibronectins to promote their contractile force. Recently, the Hippo pathway was shown to contribute to the pathogenesis of fibrosis, in which hyperactive YAP/TAZ accumulate in both the epithelial and stromal tissue compartments of fibrotic tissues.

### 2.1. Lung Fibrosis

The basic unit of the lungs is the pulmonary alveoli comprised of epithelial and ECM layers surrounded by capillaries [33]. During lung development and repair, respiratory epithelial and mesenchymal progenitors show dramatic changes in their cellular behavior to maintain the appropriate cell type composition and structural organization of the lung epithelium through a re-epithelialization process [34]. The alveolar epithelial cells are the main source of fibroblasts and myofibroblasts, which convert to a mesenchymal cell phenotype following injury, and could trigger the development of fibrosis through a process known as epithelial-mesenchymal transition (EMT). The Hippo pathway controls epithelial progenitor cell proliferation, migration, and differentiation in the developing and mature lungs [35,36].

TAZ is proposed to act as a co-activator of various transcriptional factors through binding of the WW domain to the (L/P)PXY motif of transcriptional factors. During fetal lung development, TAZ and thyroid transcription factor 1 (TTF-1) are co-expressed in respiratory epithelial cells, and TAZ directly interacts with the (L/P)PXY motif of TTF-1 to activate the transcription of target genes, including surfactant protein C (SP-C) that maintains lung morphogenesis [37]. Moreover, Mitani et al. [38] showed that TAZ knockout mice exhibited abnormal lung alveolarization. Microarray analysis of wild-type and TAZ-deficient mouse lungs revealed that connective tissue growth factor (*Ctgf*), a known direct TAZ-TEAD target gene, is the main player in peripheral epithelial cell differentiation and lung development. Previous studies have also suggested that CTGF promotes EMT, lung fibrosis, and lung development, implying a correlation between TAZ and CTGF in lung physiological and pathological functions [39,40,41].

**Figure 1:**
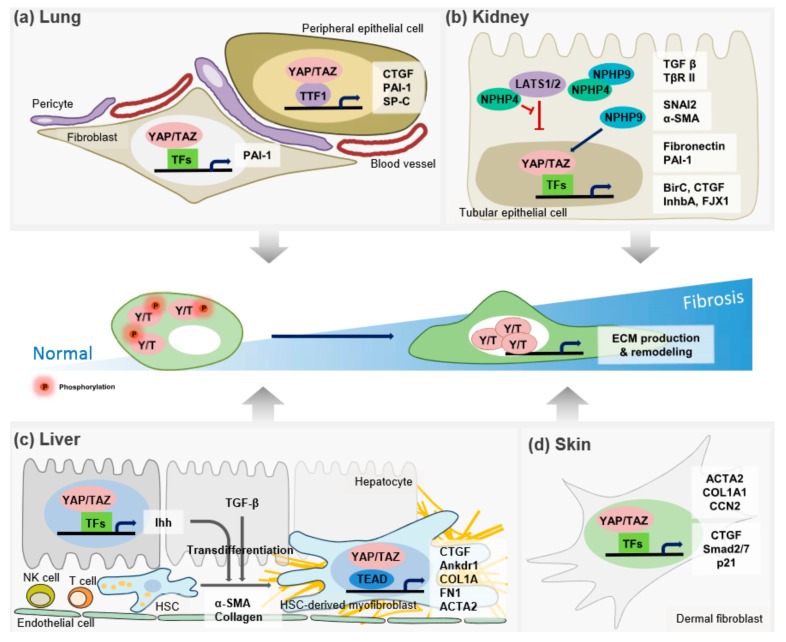
The role of the Hippo pathway in the pathogenesis of tissue fibrosis. In normal cells, YAP/TAZ are phosphorylated and localized in the cytoplasm, leading to their inactivation. However, in many pathological situations, YAP/TAZ localize to the nucleus, and up-regulate their target genes to remodel the ECM. This action leads to tissue fibrosis. (**a**) In respiratory epithelial cells, binding YAP/TAZ with TTF-1 synergistically activates the expression of target genes, including SP-C, CTGF, and PAI-1. Also, active fibroblasts promote matrix synthesis via YAP/TAZ that are critical to the induction of profibrotic genes, such as CTGF and PAI-1, in the development of lung fibrosis. (**b**) NPHP4, by interacting with LATS1/2, promotes YAP/TAZ transcriptional activity. In contrast, NPHP9, another NPH proteins (NPHPs), directly interacts with YAP/TAZ and induces its nuclear localization to regulate a subset of target genes involved in renal fibrosis. (**c**) YAP/TAZ-mediated Indian hedgehog *(Ihh)* gene induction, which increases transcriptional reprogramming by modulating profibrotic gene expression in HSCs. In response to Ihh and TGF-β, HSCs transdifferentiate into the ECM-producing myofibroblasts. (**d**) YAP/TAZ are mainly localized with the nucleus, resulting in the profibrotic gene expression in dermal fibrolasts. See text for further details. The red spheres indicate the phosphorylation of YAP/TAZ by kinase.

Moreover, the matrix mechanical environment influences the subcellular localization of YAP/TAZ in lung fibroblasts [42]. An active YAP/TAZ positive feed-forward mechanism was shown to amplify a profibrotic response to induce fibrosis in the lung tissue of mice following tail vein injection of YAP 5SA/TAZ 4SA-overexpressing fibroblasts. In a similar fashion, YAP and TAZ were found to be highly expressed in the fibroblastic foci of the lungs from patients with idiopathic pulmonary fibrosis (IPF) with obvious nuclear expression of YAP/TAZ, indicating that YAP/TAZ play a significant role in fibrosis [43]. When cultured on a stiff matrix, YAP/TAZ accumulated in the nucleus, and the lung fibroblasts exhibited increased proliferation, contraction, and ECM production, comparable to the results for cells cultured on a soft matrix. However, silencing of YAP and TAZ by small interfering RNAs (siRNAs) reduced the fibroblast responses induced by a stiff matrix, suggesting that YAP and TAZ activation are indispensable for these phenotypic features of myofibroblasts. In further support of the association of the function of YAP/TAZ with lung fibrosis, Liu et al. showed that the forced expression of a constitutively active form of YAP and TAZ in lung fibroblasts increased the expression of CTGF and PAI-1, also known as SERPINE1, along with that of ECM-related proteins such as collagens and fibronectin on a stiff matrix. However, an active TAZ mutant could not induce the expression of these ECM proteins in cells cultured on a soft matrix. These findings suggested that both mechanical stress and fully activated TAZ are necessary to induce the expression of profibrotic genes in the development of tissue fibrosis [44]. RNA sequencing identified that the genes that are positively regulated by TAZ in lung fibroblasts are linked to cell migration and motility, and are closely related to genes regulated by transforming growth factor (TGF)-β, a central mediator in lung fibrosis [43]. In addition, the profibrotic effect of YAP/TAZ was demonstrated in murine models of pulmonary fibrosis. Deletion of a single copy of *Taz* (WWTR1) attenuated bleomycin-induced pulmonary fibrosis and diminished collagen deposition and lung elastance, indicating that TAZ is a key molecule in fibrogenic events [38]. More recently, emerging evidence suggests the involvement of microRNA (miRNA) in IPF progression. For example, YAP1/Twist-induced fibroblast activation and fibrogenesis were blocked by miR15a [45]. IPF is a progressive chronic interstitial lung disease and is usually a fatal condition with no available cure [46]. IPF is characterized by fibroblast proliferation, remodeling of the ECM, and the contraction of fibroblasts, which contribute to tissue tension or stiffness that results in irreversible distortion of the lung architecture [47]. Single-cell RNA profiles from both normal and IPF lung epithelial cells demonstrated that gene expression patterns of IPF cells were in a multilineage-like state, which was not detected during normal lung development [48]. Transcriptomic analysis of IPF cells identified abnormal activation of several pathways that regulate the multilineage differentiation program, including TGF-β, Hippo, PI3K/AKT, p53, and Wnt signaling cascades. Nuclear YAP localization was shown to be increased in IPF lung epithelial cells [49]. YAP cooperates with mTOR-PI3K-AKT signaling to activate aberrant cell proliferation and migration, and inhibits the epithelial cell differentiation that may be involved in the development of IPF. Thus, mechanistically, YAP/TAZ activity contributes to the pathogenesis of IPF in both lung fibroblasts and epithelial cells. Indeed, Speight et al. showed that alpha-smooth muscle actin (α-SMA) expression is regulated by TAZ in the injured epithelium during phenotypic changes such as the epithelial–myofibroblast transition [50]. Similarly, recent reports demonstrated that stiff matrices activated fibroblasts and hepatic stellate cells (HSCs) to promote matrix synthesis through YAP/TAZ-mediated gene induction, including *CTGF* and *PAI-1* [35,43]. Taken together, these observations suggest that YAP and TAZ are important mechanosensors of TGF-β-dependent and -independent lung fibrogenesis (Figure 1a).

### 2.2. Kidney Fibrosis

The kidney comprises numerous distinct cell types that are organized into a renal filter, including glomerular endothelial cells, glomerular basement membrane, and podocytes [51]. In mice, YAP/TAZ are essential for epithelial cell growth and establishing the proper kidney architecture [52,53,54]. Several studies have demonstrated the involvement of YAP/TAZ in the progression of cystic kidney disease (CKD) in patients and in a mouse model based on their enhanced nuclear localization in epithelial cells [55,56,57]. Various renal injuries resulting from IgA nephropathy or membranous nephropathy lead to renal tubulointerstitial fibrosis (TIF), which is the end stage of kidney failure known as progressive fibrotic chronic kidney disease. TAZ protein levels were shown to be markedly increased in both mouse *Sav1*-depleted kidneys after unilateral ureteral obstruction (UUO) and in patients with TIF/CKD [58]. High-level expression and nuclear accumulation of TAZ in the renal tubulointerstitium were demonstrated in three mouse models of renal injury [59]. Consistent with these findings, the YAP protein levels increased in both the cytoplasm and nucleus in regenerative and poorly differentiated renal tubules during the transition from acute kidney injury to CKD [60]. In addition, activation of YAP/TAZ after UUO led to TGF-β-induced EMT-like features in renal TIF [58]. As expected, deletion of *Sav1* in renal tubular epithelial cells induced YAP/TAZ activation and increased the EMT phenotype via induction of SNAI2 and a-SMA, which may improve renal fibrosis.

Other studies have demonstrated that the Hippo pathway plays a crucial role in tissue repair after renal injury and is involved in maintaining the kidney filtration barrier (Figure 1b) [53,61,62]. Podocyte-specific deletion of *Yap1* in mice induced cell death and the progression of significant proteinuria and glomerular lesions [63]. In kidney fibrosis, TGF-β-induced profibrotic Smad signaling by controlling Smad2/3 localization is regulated by stiffening of the ECM, and this mechanosensing is mediated by YAP/TAZ in renal fibroblasts [64]. The abundance and activation of YAP/TAZ were detected in podocytes of a puromycin aminonucleoside-induced injury model of glomerular disease [62]. Gene expression analysis in the podocytes further indicated that overexpressed YAP upregulated ECM constituents (i.e., COL6A1, BCAM, and ADAMTS1) and growth factor-related proteins (i.e., PLOD2, PDAP1, and PRMT5), which changed the mechanical properties of the glomerular basement membrane, a prognostic marker of glomerular disease progression.

### 2.3. Liver Fibrosis

The Hippo pathway also plays an important role in liver development [65]. *Yap* overexpression or *Mst1/2*-knockout in the liver and other upstream regulators causing hyperactivation of YAP was shown to contribute to hepatomegaly and liver tumorigenesis [14,66,67,68]. Large livers comprise activated progenitor cells, hyperproliferating hepatocytes, and bile duct cells, and are more likely to develop liver cancer at later stages [14,66,68]. Thus, the Hippo signaling pathway may control the activation of progenitor cells and their differentiation towards the hepatocyte lineage. HSCs are the predominant mediators of liver fibrosis/cirrhosis and are rapidly activated and transdifferentiate into fibrogenic hepatic myofibroblasts during liver injury. HSCs from a CCl4-induced mouse liver fibrosis model and from the livers of patients with hepatitis C virus infection showed predominant nuclear YAP localization, which could be used for staging fibrosis [69]. High abundance of nuclear YAP denotes the activation of YAP in myofibroblasts. YAP inhibition by specific siRNA or treatment with verteporfin (VP), which disrupts the YAP/TAZ-TEAD interaction, decreased target gene expression and prevented the transdifferentiation of quiescent HSCs into myofibroblasts. YAP has been confirmed as a critical driver of HSC activation based on studies with in vivo and in vitro models of liver fibrosis. Consistent with this concept, Hedgehog signaling-mediated YAP activation was found to be necessary for HSC transdifferentiation and proliferation by a glutaminolytic process [70]. TAZ expression is elevated in hepatocytes of the liver of patients with non-alcoholic steatohepatitis (NASH) and in NASH model mice [71].

The key mechanism driving the transition from steatosis to NASH is TAZ-mediated Indian hedgehog (*Ihh*) gene induction, which enhances transcriptional reprogramming by modulating profibrotic gene expression in HSCs, the fibrogenic precursor cells of ECM-producing myofibroblasts. In the same study, treatment of TAZ-specific siRNA to the hepatocytes isolated from NASH mouse model suppressed hepatic inflammation, fibrosis, and cell death, whereas reconstituted TAZ expression promoted the progression from steatosis to NASH (Figure 1c).

### 2.4. Heart Fibrosis

The adult mammalian heart has restricted regenerative capacity and repair potential. Thus, after injury or disease, a large number of cardiomyocytes are lost and a fibrotic scar is formed, which can result in heart failure [72]. Conversely, the neonatal heart has potential for enhanced cardiomyocyte proliferation. Therefore, elucidation of the regulatory mechanisms underlying cardiomyocyte proliferation may provide new insight for regenerative repair strategies. Several studies have also demonstrated a role of the Hippo pathway in regulating cardiac development. During mouse embryogenesis, loss of *Mst1/2* or *Lats*, parts of the central kinase cascade, induced cardiomyocyte proliferation [73]. Similarly, in damaged hearts, inactivated *Salv* and *Lats* led to re-entry of the mitotic cell cycle and subsequent cytokinesis, indicating that Hippo signaling is a major regulator of cardiomyocyte renewal and regeneration [74]. A constitutively active S112A mutant form of YAP enhanced cardiomyocyte proliferation and heart growth, whereas cardiac-specific deletion of *Yap* decreased cardiomyocyte proliferation in the embryonic and postnatal mouse heart [75,76]. Consistent with these findings, deletion of *Yap* and *Taz* was shown to result in lethal cardiomyopathy, whereas activated *Yap* stimulated cardiac regeneration and enhanced contractility after myocardial infarction in the adult mouse heart [77]. Insulin-like growth factor signaling and the Wnt pathway cooperatively contribute to the beneficial effects of YAP on fetal cardiac growth, homeostasis, and normal function of the postnatal heart [73,75,78]. In addition, Angiotensin II (ANG II) induced cardiovascular fibrosis in a YAP/TAZ-dependent manner. Lovastatin mediated YAP/TAZ suppression decreased ANG II-induced fibrogenic gene expression as well as cardiovascular fibrosis [79].

### 2.5. Skin Fibrosis

YAP also plays an important role in maintaining normal skin homeostasis in response to extracellular cues by promoting cell proliferation in epidermal stem and progenitor cells, and tissue expansion [80,81] Genetic analysis showed that YAP is highly expressed and shows nuclear localization in epidermal progenitor and stem cells, revealing a requirement for TEAD to mediate YAP’s functions in controlling their self-renewal and maintaining their undifferentiated state [82]. Moreover, enhanced nuclear YAP/TAZ levels have been detected in the dermis during the skin wound-healing process. Downregulation of YAP/TAZ expression delayed healing and reduced the expression levels of TGF-β and its target genes such as p21 and Smad family members [83]. The expression and activation of YAZ/TAZ are increased in Dupuytren disease tissues, which are characterized by the formation of myofibroblast-rich cords and nodules in the hands [84]. This study further showed that YAP1 is involved in the differentiation of human dermal fibroblasts to myofibroblasts, and is necessary for maintenance of a smooth muscle cell-like contractile phenotype in primary Dupuytren myofibroblasts. In addition, YAP/TAZ levels were found to be elevated in systemic sclerosis (SSc) biopsy sections, mainly localized within the nucleus, and they mediated the profibrotic responses in dermal fibroblasts [85]. In mice, depletion of YAP/TAZ was effective in inhibiting bleomycin-induced fibrosis, a model of SSc dermal fibrosis, via inhibition of the PI3K-AKT-GSK3-β pathway. Mechanistically, dimethyl fumarate significantly blocked nuclear YAP localization in SSc dermal fibroblasts and prevented bleomycin-induced skin fibrosis (Figure 1d).

### 2.6. Tumor Fibrosis

In normal conditions, myofibroblasts modulate the wound-healing process and participate in tissue remodeling via producing a large amount of ECM, matrix metalloproteinases (MMPs), and tissue inhibitors of metalloproteinases (TIMPs) [86]. Myofibroblast activation ultimately occurs via MMPs and TIMPs-mediated ECM reorganization and contraction, conferring the contractile properties of myofibroblasts containing contractile actin filament bundles. In solid tumors, cancer cells reciprocally interact with non-cancerous stromal cells and the ECM to create a permissive tumor microenvironment for proliferation and maintenance of cancer cell-like features [87,88,89,90]. In particular, fibroblasts that generally reside in the tumor microenvironment are denoted as cancer-associated fibroblasts (CAFs). During tumorigenesis, both cancer cells and infiltrated immune cells release various cytokines, growth factors, and mediators to stimulate the transdifferentiation and activation of CAFs [91]. CAFs were also suggested to facilitate cancer cell invasion and metastasis through the production of soluble factors and remodeling of the ECM. A co-culture model of cancer cells and stromal fibroblasts revealed that both protease and force-mediated ECM remodeling enabled the invasion of cancer cells via a mechanism requiring CAFs [92]. CAFs mainly promote cancer cell invasion by altering the organization and physical properties of the basement membrane, leading to the formation of gaps that allow for migration [93]. For instance, mRNA expression profiling analyses showed that YAP activation is critical for the establishment and maintenance of CAFs, and that its function is necessary for ECM stiffness modulation in the progression of mammary tumors [94]. YAP/TAZ upregulation is a representative marker of high-grade breast cancer, which is required for CAFs to induce ECM stiffening, angiogenesis, and invasion of cancer cells [95]. Remarkably, increased ECM stiffness promoted cellular YAP activity to induce its target genes, and therefore support their maintenance in the tumor microenvironment by regulating matrix stiffening. Furthermore, ECM stiffness and YAP/TAZ activity function via a reinforcing feed-forward loop to preserve the CAF phenotype. Strikingly, the MRTF-SRF pathway is activated in CAFs and is required for its contractility and pro-invasive properties [96]. Activation of the MRTF-SRF pathway, which is also mechano-responsive and mandatory for maintenance of the CAF phenotype, is dependent on the transcription co-activator YAP. MRTF and YAP can indirectly activate each other through their ability to modulate actin cytoskeletal dynamics and tumor invasion under control of Rho GTPase signaling.

### 2.7. The Links between Fibrosis and Cancer via the Hippo Pathway

**Figure 2:**
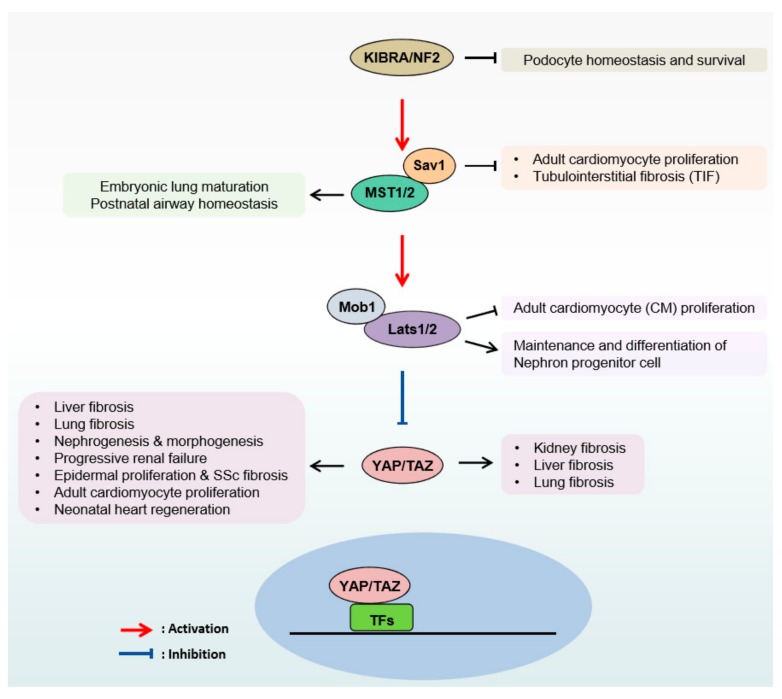
The Hippo pathway drives fibrosis across the organs.

The wound-healing process in injured tissue results in the infiltration of stromal cells, including immune cells, endothelial cells, pericytes, adipocytes, and fibroblasts, to the local tissue. This results in the release of cytokines and growth factor from infiltrated immune cells, which triggers the activation and transdifferentiation of fibroblasts into myofibroblasts. Upon injury, changes to the microenvironment activate myofibroblasts to secrete several growth factors, chemokines, and ECM components, including fibronectin, collagens, laminin, fibronectin, and tenascin [86,97]. In the case of a chronic injury that causes long-term inflammation, myofibroblasts rapidly increase the production of ECM, resulting in excessive deposition and resultant fibrosis. Stressed myoblasts are key actors in fibrosis development, and act as drivers in metastasis and invasion during tumor progression, thus representing the main link between fibrosis and cancer development [91,98,99,100]. Fibroblast-mediated ECM changes in the stroma may trigger a dramatic alteration in the Hippo pathway in both fibrosis and tumor progression (Figure 2). Recent findings imply that ECM cues and YAP/TAZ collaborate to operate a positive feedback circuit in cancer cells that contributes to matrix stiffening, resulting in further activation of YAP/TAZ oncogenic activity.

## 3. The Hippo Pathway in Cancer Progression

### 3.1. Genetic and Epigenetic Altherations in the Hippo Pathway Genes

Actively proliferating cells or cancer cells typically show a common feature of highly enriched YAP/TAZ protein in the nucleus, which exists in a dephosphorylated form and can enhance fundamental cellular functions, including cell proliferation and tumorigenesis [101]. Extensive research implicates dysregulation of the Hippo pathway in a wide spectrum of human cancers across the body [7,102]. However, only limited cancers have been associated with somatic mutations in components of the Hippo pathway compared with those of key growth and oncogenic signaling pathways. For instance, NF2, an upstream regulator of the Hippo pathway, is an extensively studied tumor suppressor, and a high frequency of NF2-inactivating mutations is a characteristic of several human cancers, including acoustic neuromas, meningiomas, schwannomas, and mesothelioma [103,104,105]. Interestingly, the core MST1/2-LATS1/2 kinase module functions as a tumor suppressor in transgenic and knockout mouse models, and no somatic mutation in these genes has been discovered in human cancer to date. However, methylation-mediated epigenetic mechanisms contribute to downregulation of LATS1/2, MST1/2, and its regulator RASSF1 in some cancer types [106,107,108]. This suggests that loss-of-function mutations in tumor suppressor genes may be mediated by non-mutational mechanisms [67,68,108,109,110,111,112]. Mutation of the *YAP* gene at the chromosome 11q22 amplicon has been reported in various human cancers [113,114]. Recently, a YAP and transcription factor E3 (TFE3) (*YAP-TFE3*) fusion gene, and a TAZ and calmodulin-binding transcription activator 1 (CAMTA1) (*TAZ-CAMTA1*) fusion gene were reported in cases of epithelioid hemangioendothelioma, a rare type of soft tissue sarcoma [115,116]. Although the effects of these fusions on the functions of the translated proteins in cancer development and progression remain to be determined, they may be functionally related to the transcriptional regulatory properties ascribed to both TAZ-CAMTA1 and YAP-TFE3.

### 3.2. YAP/TAZ as Mediators of the Mechanical Cues Shaping the Tumor Microenvironment

To respond to changes in the extracellular environment, cells harmonize an intracellular signaling network to achieve the desired cellular behavior during physiological growth and regeneration. ECM mechanical cues are powerful determinants of cell proliferation, differentiation, and cell death, acting as potent regulators of YAP/TAZ. For instance, the cell-cell contact resulting from a high cell density activates the growth inhibition signaling pathway that is predominantly mediated by the Hippo pathway [22,117,118]. As the cell density increases, LATS1/2 are phosphorylated and activated by cell-to-cell contact, leading to the inactivation of YAP/TAZ, which constitutes a central mechanism for contact inhibition. This contact-mediated inhibition in turn regulates YAP/TAZ-TEAD-mediated transcription, which is also important for embryo development [119]. Dupont et al. [120] first reported that the intracellular localization and activity of YAP/TAZ correlate with ECM rigidity and cell spreading. For example, YAP/TAZ translocate to the nucleus and function as transcriptional co-activators in response to exposure to a stiff ECM, and can stimulate the proliferation and survival of cancer cells. However, on soft matrices, cells are rounded and minimally adhesive by limiting the adhesive area. As expected, the main subcellular localization of YAP/TAZ is the cytoplasm. YAP/TAZ are downstream effectors of RhoA-ROCK-mediated actin cytoskeleton rearrangements, and thus regulate development and tumorigenic processes. In particular, a comprehensive study suggested that LATS1/2-dependent and -independent mechanisms synergize mechanical cues mediated by YAP/TAZ activation. When a cell detaches from the ECM, it undergoes the apoptotic process of anoikis owing to a dysregulated cytoskeleton and increased LATS1/2 kinase activity, which in turn inhibits YAP/TAZ activity [121].

**Figure 3:**
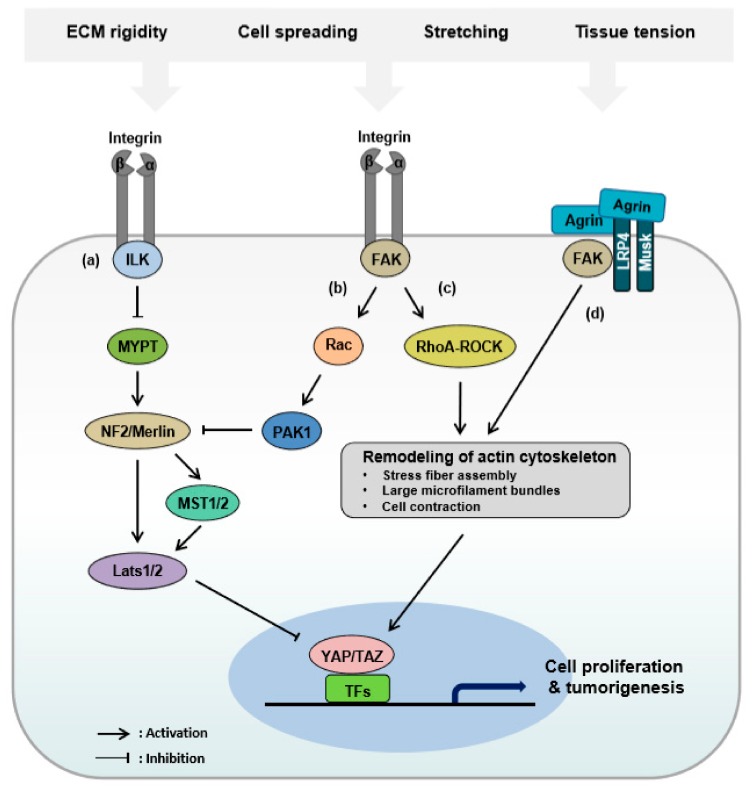
Schematic model of regulation of the Hippo pathway by numerous signaling pathways in cancer. (**a**) Integrin-ILK signaling inhibits MYPT1, leading to inactivation of the MST1/2-LATS1/2 kinase cascade, which triggers YAP/TAZ activation. (**b**) RAC1 and PAK1 phosphorylate NF2 that inhibits the interaction between YAP and NF2, resulting in YAP/TAZ activation. (**c**) Integrin-FAK signaling activates the RhoA-ROCK pathway to control remodeling of the actin cytoskeleton. (**d**) In response to ECM, Agrin enhances RhoA-ROCK-dependent actin cytoskeletal remodeling. As a later event, YAP/TAZ are translocated to the nucleus to interact with transcription factors (TFs), which drive profibrotic and tumorigenic effects.

Furthermore, Hippo pathway activity and YAP/TAZ localization depend on cytoskeletal dynamics in response to a mechanical input, including cell spreading, stretching, size, and shape [120,122,123]. Integrin-mediated cell adhesion to the ECM and association with intracellular actin filaments is required for many physiological functions [124]. Focal adhesions comprising focal adhesion kinase (FAK), Src family kinases, and integrins act as a sensor of the physical properties of the environment surrounding the ECM through forces exerted on the adhesions and via intracellular signals in response to extracellular physiochemical stimuli. FAK activates the RhoA-ROCK signaling pathway that controls remodeling of the actin cytoskeleton, which increases the stress fiber assembly, large microfilament bundles, and cell contractility (Figure 3c). In addition to FAK, ILK has been implicated in integrin-mediated signaling and promotes the inhibitory phosphorylation of MYPT1, leading to inactivation of Merlin, which is a key upstream regulator of the Hippo pathway (Figure 3a) [125]. This integrin-mediated FAK–Src signaling triggers YAP/TAZ activation. Moreover, integrin-dependent cell adhesion induces p21-activated kinase 1 (PAK1) activation, which is required for YAP/TAZ nuclear localization in a Merlin-dependent manner (Figure 3b). The small GTPase RAC1 and its downstream effector PAK1 promote the direct phosphorylation of NF2, which potentiates the physical interaction between NF2 and YAP, resulting in the nuclear translocation of activated YAP/TAZ [126]. In addition, fibronectin adhesion-induced stimulation of the FAK-Src-PI3K pathway acts as an upstream negative regulator of the Hippo pathway, which leads to activation of YAP/TAZ in a LATS1/2 kinase-dependent manner [127]. Interestingly, the surrounding ECM activates YAP/TAZ directly to promote the transcription of genes involved in cytoskeleton remodeling through the RhoA-ROCK pathway [128].

Morphological manipulation without cell–cell contact and changes in the number of stress fibers according to the adhesion area enhances the nuclear localization of YAP through activation of the Hippo pathway [129]. Moreover, deficiency of destrin, an actin-depolymerizing factor, leads to the induction of F-actin polymerization and the consequent hyperproliferation of corneal epithelial cells [130]. In contrast, reduction of the F-actin binding protein Cofilin, CapZ, and gesolin, which are essential elements of the actin cytoskeleton, aberrantly activates F-actin polymerization and therefore restricts activation of the Hippo pathway to in turn positively regulate YAP/TAZ activity [123]. Agrin, a proteoglycan, promotes liver carcinogenesis as an ECM sensor, leading to enhancement of YAP stability and activity through the combinatory activation of integrin-FAK- and Lrp4/MuSK receptor-mediated signaling pathways (Figure 3d). In this context, Agrin transduces stiffness signals via RhoA-dependent actin cytoskeletal rearrangements. Stimulated ILK-PAK1 signaling antagonizes Merlin and LATS1/2 activation [131,132,133]. Glypican-3, a member of the glypican family of heparan sulfate proteoglycans, is highly and specifically expressed in hepatocellular carcinoma (HCC) [134]. Glypican-3 targeting HN3 was shown to significantly inhibit the growth of HCC cells and xenograft tumors in nude mice by inhibiting YAP function. Disturbed fluid flow is widely recognized to induce the expression of YAP/TAZ target genes through the coordination of Rho GTPase activities, highlighting YAP/TAZ as a key node in mediating mechanical cues and maintaining vascular homeostasis [135,136,137]. Moreover, the mechanical stability of the nucleus dictates YAP nuclear entry [138], and a mechano-sensing mechanism has been identified to be directly mediated by nuclear pore complexes. On a stiff substrate, focal adhesions and stress fibers lead to nuclear flattening, which stretches the nuclear pores by transducing mechanical forces. These stretched and curved nuclear pores induce YAP import due to the relaxation of mechanical restriction to nuclear pore complexes.

**Figure 4:**
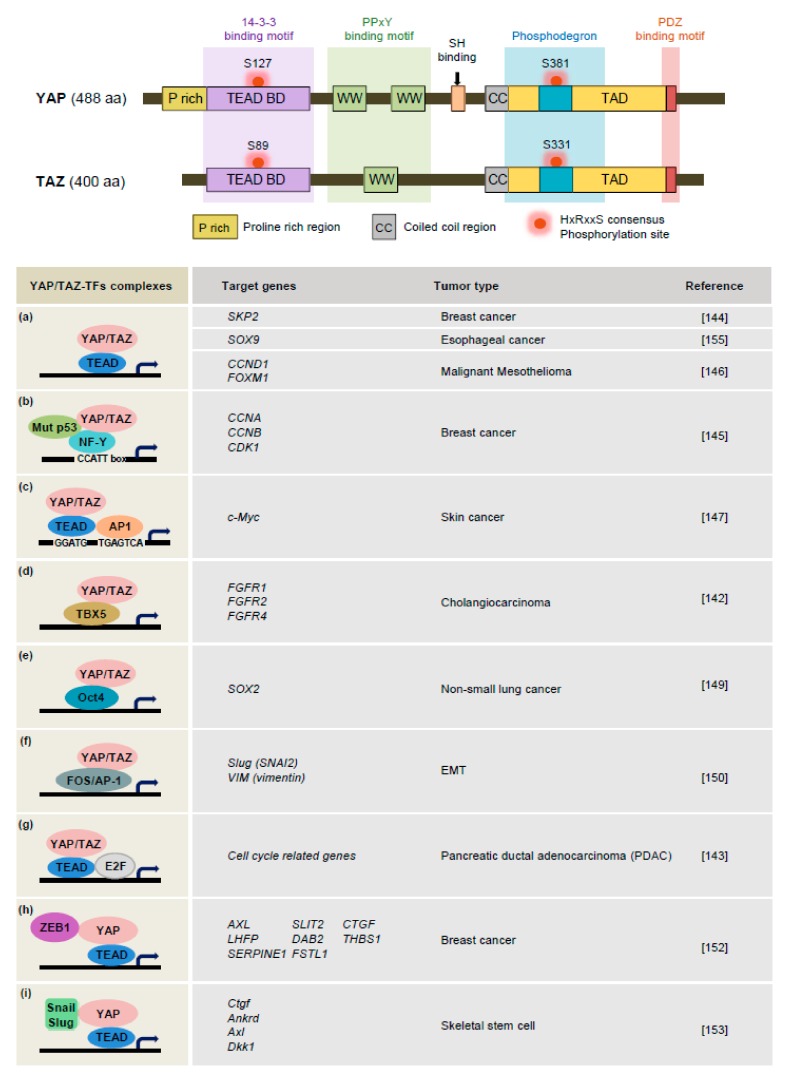
YAP/TAZ-mediated regulatory networks in cancer. YAP/TAZ are the major downstream mediators of the Hippo pathway and are composed of several domains (see Figure 1). The nuclear YAP/TAZ interacts with multiple transcription factors and signaling pathway that collectively contribute to tumorigenesis.

Overall, Rho GTPase and cytoskeletal tension are responsible for the transduction of mechanical stress in cells and have substantial effects on YAP/TAZ activity. For example, an F-actin disruptor and Rho GTPase inhibitor considerably affect YAP nuclear translocation and activity [120,121,129]. However, it remains unclear precisely how the actin cytoskeleton and mechanical stress converge on YAP/TAZ regulation. Detachment of cells from the ECM triggers LATS1/2, lead to inactivation of YAP/TAZ in a cytoskeleton- or JNK-dependent manner [129,139]. Accordingly, the detailed mechanisms underlying involvement of the Hippo pathway kinases MST1/2 and LATS1/2 as mediators to connect mechanical cue remain to be fully elucidated. Beyond ECM remodeling, CAFs are also capable of secreting a wide range of mediators, including growth factors, chemokines, MMPs, TIMPs, and ECM components [100]. These secreted mediators can act as ligands for receptors expressed by other cell types in the tumor microenvironment, which initiate the signaling cascades that activate YAP/TAZ and lead to tumor progression. For example, in response to epidermal growth factor, its receptor regulates the Hippo pathway through the PI3K-PDK1 pathway, resulting in inactivation of LATS1/2 and activation of YAP [140]. Vascular endothelial growth factor, a major driver of blood vessel formation, activates YAP/TAZ via actin cytoskeleton dynamics to facilitate a transcriptional program to further control cytoskeleton dynamics and initiate a proper angiogenic response [141]. Fibroblast growth factor 5-driven fibroblast growth factor receptor (FGFR) signaling promotes YAP activation, setting off a feed-forward loop whereby YAP forms a complex with TBX5 and induces the expression of FGFR1, 2, and 4 in human cholangiocarcinoma cell lines (Figure 4d) [142]. YAP and KRAS coordinately regulate the cell cycle and DNA replication program by forming a complex with the transcription factor E2F in pancreatic ductal adenocarcinoma (Figure 4g) [143]. Jang et al. (Figure 4a) [144] showed that mechanical stress regulates the cell cycle via the Hippo pathway. In breast cancer cells, YAP and TEAD directly bind to the *Skp2* promoter to enhance its transcription upon receiving a mechanical cue, which is necessary for cell cycle progression. Interaction of YAP with mutant p53 together form a complex with NF-Y, a nuclear transcription factor, which upregulates the expression of cell cycle genes, including cyclin A (*CCNA*), cyclin B (*CCNB*), and *CDK1*, in breast cancer cells (Figure 4b) [145]. YAP also enhances malignant mesothelioma cell growth and survival via the upregulation of cyclin D1 (CCND1) and FOXM1 in cooperation with TEAD (Figure 4a) [146]. Furthermore, YAP/TAZ/TEAD cooperate with AP-1 in the transcriptional amplification of the c-Myc oncogene to promote cell cycle progression in skin cancer (Figure 4c) [147].

### 3.3. YAP/TAZ Signaling in the EMT

The EMT is a complex transdifferentiation process that allows for epithelial cells to acquire the stem-like properties of mesenchymal cells, and is an important process in both fibrosis and cancer metastasis. The EMT is executed through a series of transcription factor networks, including TWIST1/2, ZEB1/2, Snail, and Slug. During cancer progression, cancer cells acquire aggressive features such as migration, invasion, chemoresistance, and apoptosis resistance that are all triggered by the EMT [148]. YAP/TAZ signaling has also been shown to play a role in the EMT in mammary epithelial cells. Constitutively active TAZ was shown to promote cell proliferation and EMT [25]. Moreover, YAP levels were found to be elevated in the stem cell-like cells derived from non-small lung cancer cells, and contributed to their self-renewal through Sox2. These effects required a physical interaction between YAP1 and Oct4 (Figure 4e) [149]. YAP and KRAS act cooperatively with the transcription factor FOS to activate Slug and vimentin-mediated EMT (Figure 4f) [150]. TAZ activity is required for the self-renewal and tumorigenesis of breast cancer cells. Twist and Snail-mediated EMT processes induce Scribble delocalization so that TAZ is released from Scribble, which stimulates the accumulation of stem-like progenitor cells in breast cancer [151]. These findings suggest that TAZ-induced EMT functions as a feed-forward mechanism of TAZ activation. The direct interaction between ZEB1 and YAP, but not TAZ, stimulated the transcription of a ZEB1/YAP target gene set that is known to promote cancer aggressiveness (Figure 4h) [152]. Indeed, this target gene set was found to be an excellent predictor of poor clinical outcomes in patients with particularly hormone receptor-negative cancer. Similarly, Snail/Slug-YAP/TAZ complexes control YAP/TAZ protein levels and the expression of target genes that modulate self-renewal, differentiation, and bone formation (Figure 4i) [153]. Therefore, crosstalk between YAP/TAZ and EMT transcription factors such as ZEB1/2, Snail/Slug, and Twist modulates the EMT programs, thereby acquiring the characteristics of malignant cancer cells. A genome-wide expression study revealed that TAZ protein levels were higher in breast cancer stem cells compared with those of differentiated breast cancer cells. A high level of TAZ was also associated with the chemoresistance and migratory potential of breast cancer stem cells [154]. The YAP-TEAD complex can directly bind to the *SOX* promoter region to upregulate *SOX9* levels and induced a cancer stem cell phenotype in esophageal cancer cells (Figure 4a) [155]. YAP and the COX2 signaling pathway coordinately regulate urothelial cancer cells with stem cell-like properties via SOX2 induction [156]. Conversely, SOX2 maintains cancer stem cells in osteoblastoma by direct inhibition of NF2 and WWC1, upstream activators of the Hippo pathway, leading to activation of YAP [157] (Figure 4).

## 4. Conclusions

Accumulating evidence has highlighted the pivotal role of the Hippo pathway and its contribution to organ and tissue homeostasis under various chemical and physical signals. The MST1/2-LATS1/2 kinase cascade incorporates various upstream inputs to regulate YAP/TAZ activity and subcellular localization so as to maintain balanced processes, including physiological growth, development, and regeneration. In recent years, increasing research efforts have focused on unmasking the physiological/pathological functions of the Hippo pathway using animal models and patients, revealing a critical role in both fibrosis and cancer, which are inextricably linked. YAP/TAZ acts as a cellular mechanosensor that induces the transdifferentiation of fibroblasts to myofibroblasts in tissue fibrosis. In response to matrix stiffness, YAP/TAZ activation drives profibrotic and tumorigenic effects. YAP/TAZ signaling is also capable of initiating the EMT via EMT-related transcription factors, and this YAP/TAZ-dependent EMT program during tumorigenesis may further stimulate stemness and tumorigenicity.

These new insights into the diverse functions of the Hippo pathway will help in discovering novel therapeutic targets for the treatment of fibrosis and cancer. For example, VP is a small molecule that modulates the YAP and TEAD interaction and could reverse fibrosis. [49,64,69]. Given the clear profibrotic effect of YAP/TAZ, such pharmacological inhibitors targeting the Hippo pathway may be beneficial for treating both fibrosis and cancer (Table 1) [69,79,158,159,160,161,162,163,164,165]. However, targeting the Hippo pathway may also cause undesired harmful effects because of its central roles in organ development, homeostasis, and regeneration. Therefore, further studies to uncover the other mechanisms or unknown molecules regulating YAP/TAZ activity are expected to offer promising therapeutic targets in fibrosis and cancer progression.

## Figures and Tables

**Table 1:** Potential targets for effective Hippo pathway regulation.

Name	Mode of Action	Tested Application	Reference
Verteporfin	Disruption of YAP/TAZ- TEAD complex	HSCs isolated from C57BL/6J mice or *Smo* flox/flox mice IPF lung epithelial cells UUO-induced renal fibrosis CCl4-induced liver fibrosis mouse model	[49,63,68]
Melatonin	Inhibiting of the expression and activation of YAP1 via binding to MT1&MT2 melatonin receptors	Bleomycin induced experimental lung fibrosis in mice	[158]
Morin	Increased expression of MST1 and Lats1 Decreased expression of YAP/TAZ	Diethylnitrosamine (Den)-induced liver fibrosis rat model Hepatic stellate cells derived form human	[159]
ω-3 PUFA	YAP/TAZ degradation in a proteasome-dependent manner	CCl4-induced liver fibrosis mouse model Hepatic stellate cells derived from human and rat	[160]
Fasudil	Inhibitory effect on Rho/ROCK signaling	Bleomycin-induced mouse lung fibrosis	[161]
Dobutamine	Induced the cytoplasmic translocation of YAP	U2OS cell line	[162]
Statin	YAP/TAZ nuclear localization and activity	Breast cancer cell lines	[163]
Lovastatin	YAP/TAZ nuclear localization and activity	CCl4-induced liver fibrosis mouse model Human aortic vascular smooth muscle cells Human kidney cells	[79]
JQ-1	Inhibitor of bromodomain-containing protein 4 (BRD4) mediated profibrotic transcription	CCl4-induced liver fibrosis mouse model Hepatic stellate cells derived from mouse	[164]
